# Ultrasonic spray coating polymer and small molecular organic film for organic light-emitting devices

**DOI:** 10.1038/srep37042

**Published:** 2016-11-22

**Authors:** Shihao Liu, Xiang Zhang, Letian Zhang, Wenfa Xie

**Affiliations:** 1 State Key Laboratory on Integrated Optoelectronics, College of Electronic Science and Engineering, Jilin University, Changchun, 130012, People’s Republic of China

## Abstract

Ultrasonic spray coating process (USCP) with high material -utilization, low manufacture costs and compatibility to streamline production has been attractive in researches on photoelectric devices. However, surface tension exists in the solvent is still a huge obstacle to realize smooth organic film for organic light emitting devices (OLEDs) by USCP. Here, high quality polymer anode buffer layer and small molecular emitting layer are successfully realized through USCP by introducing extra-low surface tension diluent and surface tension control method. The introduction of low surface tension methyl alcohol is beneficial to the formation of poly (3,4-ethylenedioxythiophene) polystyrene sulfonate (PEDOT:PSS) films and brings obvious phase separation and improved conductivity to PEDOT:PSS film. Besides, a surface tension control method, in which new stable tension equilibrium is built at the border of wetting layer, is proposed to eliminate the effect of surface tension during the solvent evaporation stage of ultrasonic spray coating the film consists of 9,9-Spirobifluoren-2-yl-diphenyl-phosphine oxide doped with 10 wt% tris [2-(p -tolyl) pyridine] iridium (III). A smooth and homogenous small molecular emitting layer without wrinkles is successfully realized. The effectiveness of the ultrasonic spray coating polymer anode buffer layer and small molecular emitting layer are also proved by introducing them in OLEDs.

The positive reactions in the consuming market signify that organic light-emitting devices (OLEDs) would be likely to become the next generation display and lighting technology in the future. Nevertheless, the high manufacture cost is still an important factor that hinders further development of the OLED industrialization before the OLED products become the mainstream in display and lighting area. At present, the common used fabrication process for OLEDs is the vacuum thermal evaporation process^1^, which should assume the primary responsibility to the high manufacture costs. Thus, to reduce the manufacture costs, solution processes which are simple and require no complicated instruments, especially spin coating process^7^, have been proposed to fabricate OLEDs. However, although spin coating has been proved to be a useful experimental technique in laboratory, its disadvantages, such as the low material utilization, the incompatibility with streamline production and a strict requirement to the viscidity of solution, are still evident^10^,^11^. In generally, the small molecular organic materials have limited solubility and small molecular mass^7^,^12^, resulting in their relatively small viscidity so that it’s hard to use them to fabricate thin films by spin coating process.

Ultrasonic spray coating process (USCP) which has a high material utilization over 90%, has drawn the attention as a new deposition technique for low-cost photoelectric^13^and it also shows the potential to form organic thin film from low viscidity organic solution. Nevertheless, to date, the reported photoelectric devices employing ultrasonic spray coating thin films are mostly organic photovoltaic, and there are only few reports on OLEDs due to their higher requirements for film quality^16^,^17^.

In the ultrasonic spray coating process, a uniform wetting layer is the precondition of a continuous organic thin film. However, the existence of surface tension in organic solution becomes a huge obstacle for the formation of smooth organic thin film in USCP. At liquid-air interfaces, the cohesion of liquid molecules to each other is much higher than the adhesion of the air molecules to the liquid molecules, giving the liquid surface the elastic tendency to contract to the minimal area^20^,^21^. Thus, a solution with high surface tension is not suitable to form a uniform wetting layer with even thickness distribution, and the high surface tension could cause the wetting layer to be shrinking as the solution evaporates^22^,^23^, easily leading to solid film with wrinkles. To obtain the smooth organic thin film employing USCP, solution with extremely low surface tension or additional surface control method must be reasonably necessary.

In the paper, extra-low surface tension diluent and a surface tension control method have been demonstrated to be useful for eliminating the effect of surface tension. Adopting the two methods, high quality polymer anode buffer layer and small molecular emitting layer are fabricated successfully by USCP. Additionally, the effectiveness of these films is also proved by the excellent performances of the corresponding OLEDs. The green devices based on the ultrasonic spray coating PEDOT:PSS films as anode buffer layer possess comparable performance in efficiency to the ones based on spin coating film, and even have better performances in luminance and efficiency roll-off. Besides, the green OLED with ultrasonic spray coating small molecular emitting layer also exhibits comparable efficiency to the vacuum thermal evaporation one.

The USCP used to fabricate the anode buffer layers and small molecular emitting layers is shown in Fig. 1. In the process, the solution is firstly atomized to micron-sized droplets by ultrasonic nozzle. And then driven by the nitrogen (N_2_) airflow, these droplets would be widely distributed on the substrate and merge together to form an ultrathin wetting layer. Finally, after the solvent is evaporated, the solute would be retained to form the target film. At the stage of solvent evaporation, the wettability of the solvent plays an important role on the formation and uniformity of the target film. Thus, the choice of low surface tension would be the guarantee of the high film quality. However, not all the suitable organic solvent would be able to completely wet the substrate and the pre-existing organic film in the fabrication of multilayer films usually possesses low surface energy, which could reduce wettability of the solvent on it. The surface tension control method, therefore, is needed to eliminate the effect of surface tension during the solvent evaporation stage. So, assisted solvent is introduced in the fabrication of emitting layer here to rebuild new stable tension equilibrium at the border of the wetting layer so that the effect of the surface tension could be controlled to some extent.

Firstly, the PEDOT: PSS film was fabricated by USCP. Since the low surface tension methyl alcohol exhibits perfect wettability on the ITO substrate, the introduction of methyl alcohol is beneficial to the formation of high quality poly(3,4-ethylenedioxythiophene) polystyrene sulfonate (PEDOT:PSS) films. Figure 2 shows the lateral static pictures of the PEDOT:PSS droplets after falling on the ITO substrate, and the droplets are the PEDOT:PSS aqueous solution mixed with the nineteen fold weight of water or methyl alcohol, respectively. It can be seen that for aqueous solution, due to the high surface tension of water, the droplet is hard to spread out and keeps at a hemisphere shape after it falls on the ITO substrate. And yet, for the solution diluted by methyl alcohol, the droplet spreads out and covers the substrate immediately, confirming the better wettability of the methyl alcohol. Additionally, since methyl alcohol has a higher saturated vapor pressure and lower boiling point than water, the former solvent could be easily removed and the solute is retained to form the target film while the latter just shrinks slowly to concentrate the solute in a small area as time goes on. Thus, methyl alcohol is chosen as the solvent to dilute PEDOT:PSS aqueous solution in USCP. Besides, the low mass fraction of the solute should be attributed to the high material utilization of the process which needs a very small amount solute to keep the film thickness in a suitable range.

To evaluate the quality of PEDOT:PSS film fabricated by ultrasonic spray coating, their surface morphology, conductivity and transmittance properties are investigated, and the spin coating films with the identical thickness of 70 nm are also fabricated from the original PEDOT:PSS aqueous solution to be the control. The atom force microscope (AFM) topography of the PEDOT:PSS films fabricated by spray and spin coating are shown in Fig. 3. It can be seen that the root-mean-square (RMS) roughness of films fabricated by USCP and spin coating is 2.75 and 1.03 nm, indicating that the film fabricated by USCP also possesses a smooth surface. Additionally, it can be also seen in Fig. 3 that there is an obvious difference in phase images. The strong and weak contrast for ultrasonic spray coating and spin coating film are observed. It indicated that PEDOT and PSS chains have a weak phase separation in the spin coating film while there is a good phase separation in the ultrasonic spray coating. This phenomenon should be attributed to the methyl alcohol introduced to dilute the aqueous solution. The methyl alcohol with a highly hydrophilic property and a high dielectric constant will interact with the hydrophilic PSS, and produce a screening effect between the PEDOT and PSS chains which would increase the likelihood of the phase separation^24^,^25^.

Furthermore, the screening effect caused by methyl alcohol might also change conductivity of the PEDOT:PSS films^24^,^25^. To investigate the conductivity of the films, the lateral and vertical conductivity characteristics of films with fixed sizes were measured and were shown in Fig. 4. It can be found that the conductivity of ultrasonic spray coating film is better than that of the spin coating one in both lateral and vertical directions and the conductive enhancement in lateral direction would be beneficial to the lateral homogeneity of charge carriers transporting in the films. The improvement of the conductivity in ultrasonic spray coating PEDOT:PSS films should be attributed to the obvious phase separation and the conformational change of PEDOT chains^24^. The existence of methyl alcohol in PEDOT:PSS solution would orient the PEDOT chains in films to linear or extended-coil structure rather than coiled structure so that the interaction between the neighboring PEDOT chains would be easier produced. These changes also bring the PEDOT:PSS films better conductive stability since the conductivity of the ultrasonic spray coating film almost remains the same while that of the spin coating one changes obviously after running the current-voltage test 20 times as shown in Fig. 4(a). Additionally, it can be seen in Fig. 4(b) that the ultrasonic spray coating film also exhibits better tolerance to extremely high electric field intensity, and its maximum current intensity could attain to a value much higher than that of the spin coating one, providing the possibility to increase the current limit of OLEDs employing it as anode buffer layer. Meanwhile, the transmittance of the PEDOT:PSS films in visible region is also studied, as shown in Fig. 4(b). It can be seen that the films fabricated by two kinds of processes are almost identical in transmittance, noting that the introduction of methyl alcohol only has a little influence on the optical property in the visible region of the films.

Secondly, the 9, 9-Spirobifluoren-2-yl-diphenyl-phosphine oxide (SPPO1):tris [2-(p-tolyl) pyridine] iridium (III) [Ir(mppy)_3_] film was fabricated by USCP. When it comes to fabricate a small molecular emitting layer on pre-coated PEDOT: PSS, the case would be different. Although toluene used as solvent for SPPO1 and Ir(mppy)_3_ has a low surface tension, it still can’t wet the PEDOT:PSS film completely like the methyl alcohol for ITO substrate. As shown in Fig. 5(a,b), after the toluene droplet containing the small molecular solute SPPO1 and Ir(mppy)_3_ falls on the substrate covered with the ultrasonic spray coating PEDOT:PSS film, the droplet could spread out immediately, but a liquid film with uniform thickness distribution is not formed after the droplet stabilizes as the lateral pictures indicate that the spread droplet possesses a thinner edge area, noting that there is still a tiny contact angle at the edge caused by the weak surface tension. When the wetting layer is formed in USCP, the existence of the weak surface tension would shape the wetting layer and attain tension equilibrium at the border of wetting layer as shown in the left picture of Fig. 5(e). As the solvent evaporates, the equilibrium could be easily broken, and then the wetting layer would start to shrink until it disappears. Due to the low solubility of small organic molecular, the solute would dissolve out of the solution and form the film, but the instability of shrinking velocity could lead to numerous wrinkles as shown in Fig. 5(c), which would hinder the application of the film in OLEDs.

To improve the quality of the small molecular film by USCP, the effect of the weak surface tension should be controlled during the solvent evaporating stage in USCP. Thus, the toluene is used as assisted solvent surrounded the substrate to control the surface tension of wetting layer as shown in Fig. 1. The introduction of assisted solvent could form new tension equilibrium^23^as shown in Fig. 5(e), in which the interfacial tension of assisted solvent could offset the effect of the interfacial tension of wetting layer. As a result of the offset effect of assisted solvent, the tension equilibrium is hard to be broken so that the wetting layer could avoid shrinking during the solvent evaporation stage. Thus, through adding the surface tension control (STC) process, a smooth small organic molecular film without wrinkles is fabricated successfully by USCP, and its fluorescence microscopic image is shown in Fig. 5(d).

Furthermore, the surface microtopography of the small organic molecular film fabricated by USCP is also investigated. The AFM topography and phase pictures of the small organic molecular film are shown in Fig. 6. It could be seen that the film by USCP possesses a smooth surface with a sub-nanometer roughness since its RMS roughness is only 0.529 nm, indicating the possibility of the application of the film to OLEDs. Besides, it could also be found in Fig. 6(b) that the film has a homogenous phase with a low contrast equal to 10° in full scale, and the low contrast should mostly be attributed to the topographic variations. The homogenous phase exhibited by the film indicates that there is no obvious phase separation between SPPO1 and Ir(mppy)_3_ in the ultrasonic spray coating film, proving the ability of the USCP to fabricate homogenous doped films.

In order to demonstrate the effectiveness of ultrasonic spray coating polymer films, the green OLEDs based on the PEDOT:PSS anode buffer layer are fabricated and their characteristics are discussed in detail. Additionally, the green OLEDs employing ultrasonic spray coating PEDOT:PSS films as anode buffer layer are marked as SY-X, while devices based on spin coating PEDOT:PSS films are fabricated for comparison and marked as SN-X, and the X here is on behalf of the value of film thickness of anode buffer layer. The thickness of ultrasonic spray coating PEDOT:PSS film used for OLEDs has been optimized to 40 nm, and the corresponding device is marked as SY-40. Meanwhile, through the experiment optimization, the spin coating PEDOT:PSS with a thickness about 30 nm has been proved to be an efficient anode buffer layer for OLEDs which is agreeing with the reported results^8^,^26^,^27^, thus, the SN-30 is used to be the control device here. These devices share similar structures: Glass substrate/ITO/PEDOT:PSS/Di-[4-(N,N-ditolylamino)-phenyl]cyclohexaneTAPC)/4,4′,4′′-tris (carbazol-9-yl)-triphenylamine (TCTA)/Emission Layer (EML)/1,3,5-Tri[(3-pyridyl)-phen-3-yl]benzene (TmPyPB)/LiF/Mg:Ag (10:1 by weight), and the EML of the green OLEDs consist of a bipolar host 4,4′-Bis(N-carbazolyl)-1,1′-biphenyl (CBP) doped with 10 wt% tris(2-phenyl-pyridine) iridium(III) [Ir(ppy)_3_]. The schematic layer structure is given in Fig. 7. In contrast, the conventional devices without any anode buffer layers are also fabricated with a thicker 30 nm TAPC hole transport layer and marked as CL-0.

The current density-voltage-luminance characteristics of the green devices are shown in Fig. 8(a). It can be found that the turn-on voltage of CL-0, SY-40 and SN-30 is all about 3.0 V, noting that these devices could all have a sufficient carrier injection. Additionally, SY-40 and SN-30 has a lower current density than that of CL-0, which should be the consequence of the introduction of PEDOT:PSS buffer layer since the current density does not decrease obviously when a more 20 nm TAPC is introduced in CL-0 (marked as CL-0+20). It could be found in the reported papers that the difference of highest occupied molecular orbital (HOMO) between PEDOT:PSS and TAPC is not distinct, and the hole mobility of PEDOT:PSS is not lower than that of TAPC^28^. Thus, the decrease of current density in PEDOT:PSS-based devices should be attributed to that the surface of the PEDOT-PSS film is dominated by PSS^31^, which would impede the transport of hole to a certain extent. Besides, although SY-40 has a thicker PEDOT:PSS film than SN-30, it still has a higher current density, which should be attributed to the better conductivity of ultrasonic spray coating PEDOT:PSS film. When it comes to the luminance, it can be also found that the maximum luminance of CL-0, SY-40 and SN-30 is 45400, 70340 and 45380 cd/m^2^, respectively. Because of the higher current density, CL-0 has a higher luminance at the low bias voltage, but the superiority in luminance is lost after the bias voltage reaches to a certain value, indicating that the unbalance of charge carriers at high bias voltages occurs more seriously in CL-0 rather than the SY-40 and SN-30 with the buffer layer. Additionally, SY-40 has a nearly 60% increase in the maximum luminance compared with that of SN-30 and CL-0. This could be attributed to higher current density of SY-40 in high bias voltages caused by the better tolerance to extreme high electric field intensity of its anode buffer layer.

With respect to efficiency, the performance of SY-40 could also be comparable to that of SN-30 and CL-0. Figure 8(b) shows the current efficiency-luminance-external quantum efficiency (EQE) characteristics of the devices. In contrast with CL-0, SY-40 and SN-30 have a better performance in efficiency and efficiency roll-off since the maximum current efficiency and EQE of CL-0, SY-40 and SN-30 is 59.1, 61.0, 67.8 cd/A and 17.1%, 17.2%, 19.3%, while the current efficiency and EQE at the luminance of 10000 cd/m^2^ is 37.5, 56.4, 50.5 cd/A and 10.9%, 15.8%, 15.7%, respectively. Obviously, SY-40 and SN-30 have a much lower efficiency roll-off than CL-0, indicating their better carrier balance and less hole-polaron quenching. Additionally, it can be seen that although SY-40 behaves slightly better in efficiency than SN-30 at high luminance, it still has a little gap in efficiency with SN-40 at the low luminance, which should be attributed to the lateral leak current of PEDOT:PSS film between adjacent electrodes of our particular ITO substrate caused by the excellent conductivity of the ultrasonic spray coating PEDOT:PSS film. Nevertheless, the lateral leak current could be eliminated with a precise limitation in film covering area and an expanding distance between adjacent electrodes. Besides, at the high luminance region, the superiority of SY-40 in efficiency and its roll-off, relative to SN-30, might be the consequence of the better lateral homogeneity of charge carrier injected from the buffer layer in SY-40.

Finally, the electroluminescent (EL) emission spectra of the devices are also discussed. The EL spectra of CL-0, SY-40 and SN-30 at the luminance of 1000 cd/m^2^ are shown in Fig. 8(c). All the devices exhibit typical emission from Ir(ppy)_3_, peak wavelength of which is at 513 nm, but it is not identical at the shoulder peak of their spectra as Fig. 8(c) indicates that the shoulder peak of SN-30 is weakest while that of SY-40 is strongest, which should be attributed to the weak microcavity effect in OLEDs. Although the ITO conductive film used as transparent anode here has a transmittance over 80%, a weak microcavity depends on the thickness of organic layers still exists in these devices, spectra of which could further be influenced evidently by the microcavity effect^32^. In contrast the emission spectra of CL-0, SY-40 and SN-30 are influenced by the microcavity effect in different degrees due to their difference in the thickness of organic layers. The total thickness of organic layers in CL-0 is 100 nm which is the conventional values used in OLEDs, resulting in that the resonance wavelength of CL-0 is about 498 nm below its peak wavelength so that the influence on its emission spectra is comparative less. Meanwhile, as the thickness of SN-30 increases to 110 nm, the resonate wavelength redshifts to approximately 510 nm, according with the photoluminescence peak wavelength of Ir(ppy)_3_, thus emissions near the peak wavelength are enhanced while others are weakened. Likewise, as the thickness of SN-30 increases to 120 nm, the resonance wavelength continues to redshift to near 533 nm so that the shoulder in the spectra of SY-40 is much stronger than that of SN-30. Additionally, to confirm the deduction above, the SY-30 and CL-0+20 (introducing a more 20 nm TAPC) which have a same thickness with SN-30 are also fabricated, and they exhibit almost identical emission spectra to those of SN-30 as estimated. Furthermore, SY-40 also exhibits a high stability on the emission spectra at different bias voltages. As Fig. 8(d) shows, the emission spectra of SY-40 keeps almost unchangeably and its CIE coordinates just change from (0.309, 0.628) at 4 V to (0.308, 0.625) at 9 V, indicating that the PEDOT:PSS films fabricated by the USCP would not affect the stability of optical property of OLEDs.

To prove the feasibility of the small molecular film by USCP to OLEDs, the green OLEDs employing the ultrasonic spray coating SPPO1 doped Ir(mppy)_3_ film as the emitting layer are fabricated successfully with structures shown in Fig. 9, and their electroluminescent characteristics are discussed in detail. Besides, the control devices employing emitting layer fabricated by thermal vacuum evaporation process with the similar structures: Glass substrate/ITO/ultrasonic spray coating PEDOT:PSS/SPPO1: 10 wt%Ir(mppy)_3_/1,3-Bis[3,5-di(pyridin-3-yl)phenyl]benzene (BmPyPhB)/LiF/Mg:Ag (10:1), are also fabricated for comparison.

The current density-voltage-luminance characteristics of the green OLEDs are investigated. It could be found in Fig. 10(a) that the turn-on voltage of ultrasonic spray coating and vacuum evaporation devices are 4.0 and 4.2 V, respectively, and the current density of the two devices at the low bias voltages is almost identical, noting that the carrier injection of these devices at low bias voltages is less affected by the fabrication processes of the emitting layers. However, at the high bias voltages, the current density of vacuum evaporation device is much higher than that of ultrasonic spray coating device, which might be attributed to that the residual solvent and dispersed oxygen that would be harmful to the transport property of organic films in high bias voltages, inevitably exist in the film fabricated by solution process. Due to the poor performance of current density at high bias voltages, the maximum luminance of ultrasonic spray coating device is only 8130 cd/m^2^, while that of vacuum evaporation device could attain to 39300 cd/m^2^. The difference between ultrasonic spray coating device and vacuum evaporation device could be eliminated to a certain extent when the USCP is carried on in an exactly low humidity and oxygen content atmosphere. In the aspect of current density and luminance, the performances of ultrasonic spray coating device is not as good as the vacuum evaporating one, but the ultrasonic spray coating device still possesses comparable efficiency. Figure 10(b) shows the current density-luminance-EQE characteristics of the green OLEDs. It can be seen that the maximum current efficiency and EQE of ultrasonic spray coating device are 24.7 cd/A and 7.2%, respectively, which is even slightly better than the performances of vacuum evaporation one (24.0 cd/A and 7.0%). The comparable electroluminescent efficiency exhibited by ultrasonic spray coating devices could prove the practicability of ultrasonic spray coating small molecular emitting layer in OLEDs. Moreover, compared to the vacuum evaporation emitting layer, the adopting of ultrasonic spray coating emitting layer doesn’t produce obvious difference in the emission spectral characteristics. The electroluminescent emission spectra of the green OLEDs based on ultrasonic spray coating and vacuum evaporation emitting layer at 1000 cd/m^2^ are shown in Fig. 10(c). It could be found clearly that both ultrasonic spray coating and vacuum evaporation devices have emission peak wavelength at about 515 nm and their spectrum distributions are almost identical. It indicated that the different processes might have slightly different effect on the refractive index of organic layer. The green OLED employing ultrasonic spray coating emitting layer also exhibits excellent stability in emission spectra with voltage, as shown in Fig. 10(d). At different luminance, the emission spectra of ultrasonic spray coating device remains almost unchanged as indicated that when the luminance increases from 36 cd/m^2^ to 7907 cd/m^2^, the CIE coordinates just change from (0.314, 0.613) to (0.317, 0.612). The excellent stability of emission spectra further confirms the effectiveness of ultrasonic spray coating small molecular emitting layer.

## Conclusion

By adopting extra-low surface tension diluent and a surface tension control method, the polymer anode buffer layer and small molecular emitting layer with high film quality are successfully fabricated. The maximum current efficiency, EQE, luminance of green OLEDs employing ultrasonic spray coating PEDOT:PSS film as anode buffer layer are 61.0 cd/A, 17.2% and 70340 cd/m^2^, respectively, and their current efficiency and EQE could still keep at 56.4 cd/A and 15.8% at the luminance of 10000 cd/m^2^, indicating that they possess comparable performance in efficiency to the ones based on spin coating film and even better in luminance and efficiency roll-off. The corresponding green OLEDs employing the ultrasonic spray coating small molecular film as emitting layer also exhibit excellent performances since the maximum luminance, current efficiency and EQE of the green OLEDs are 8130 cd/m^2^, 24.7 cd/A and 7.2%, respectively, which is comparable to the one based on vacuum thermal evaporation emitting layer. The excellent performances exhibited by their corresponding devices confirm the effectiveness of the spray coating films. This work may be helpful to the realization of efficient solution-process small molecular OLEDs and development of low-cost OLED products.

## Methods

### Device fabrication

The small molecular organic materials used in our experiments are purchased from Luminescence Technology Corporation. All devices are fabricated on ITO glass substrates which are subjected to a routine cleaning process with rinsing in Decon 90, deionized water, drying in an oven, and finally treating in a plasma cleaner chamber. The PEDOT:PSS (Baytron CH-8000 from Xi’an p-OLED) film are fabricated by ultrasonic spray coating from a diluted 1:19 solution in methyl alcohol or by spin coating from a original aqueous solution in an nitrogen atmosphere glovebox, and then the PEDOT:PSS films are all annealed at 120 °C for 30 min. The small molecular emitting layers are also fabricated by ultrasonic spray coating from the toluene solution of SPPO1 doped with Ir(mppy)_3_, while the mass fraction is 1 mg/mL and the mass ratio of SPPO1 and Ir(mppy)_3_ is 10:1. Other emitting layers, carrier transfer layers and cathode are evaporated by thermal evaporation under high vacuum (~ 5 × 10^−4^ Pa) at a rate of 1–2 Å s^−1^ monitored *in situ* with a quartz oscillator. A shadow mask is used to define the anode and cathode, and to make four 10 mm^2^ devices on each substrate.

### Device characterization

The static lateral images are measured using a contact angle goniometer (JC2000D contact Angle Meter, Powereach Co. Shanghai, China). The morphologies of the ultrasonic spray coating films are characterized by using atomic force microscopy (AFM, Dimension Icon, Bruker Co.) in contact mode, and the thickness of ultrasonic spray coating films are obtained by an ellipsometer (J. A. Woollam Co. Inc). Luminance-current-voltage characteristics and spectra of unpackaged devices are measured simultaneously using Goniophotometric Measurement System based on spectrometer (GP-500, Otsuka Electronics Co. Osaka, Japan) in air at room temperature.

## Additional Information

**How to cite this article**: Liu, S. *et al*. Ultrasonic spray coating polymer and small molecular organic film for organic light-emitting devices. *Sci. Rep.* **6**, 37042; doi: 10.1038/srep37042 (2016).

**Publisher's note**: Springer Nature remains neutral with regard to jurisdictional claims in published maps and institutional affiliations.

## Figures and Tables

**Figure 1:**
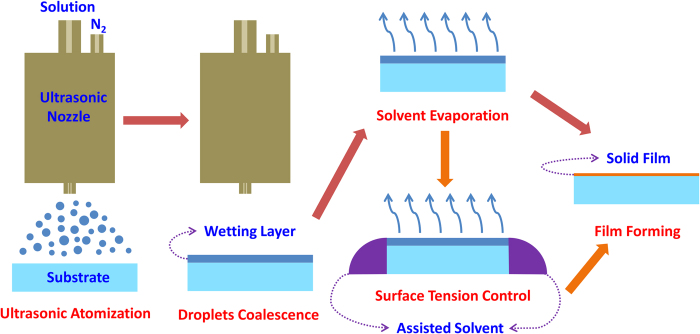


**Figure 2:**
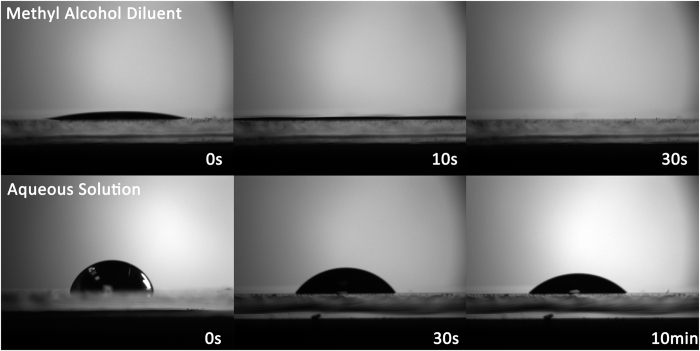


**Figure 3:**
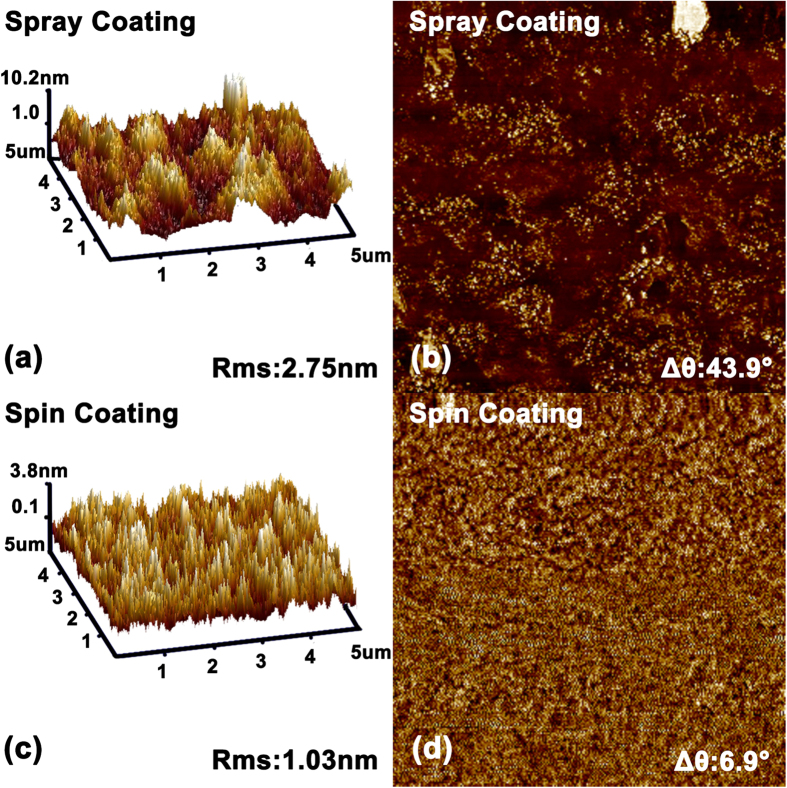
AFM (**a**,**c**) topography (5 μm × 5 μm) and (**b**,**d**) phase pictures of PEDOT:PSS films fabricated by spray and spin coating process.

**Figure 4:**
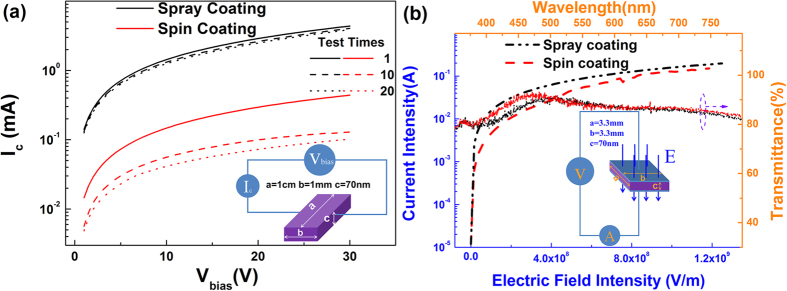
(**a**) The lateral conductivity, (**b**) the vertical conductivity and transmittance characteristics of PEDOT:PSS films.

**Figure 5:**
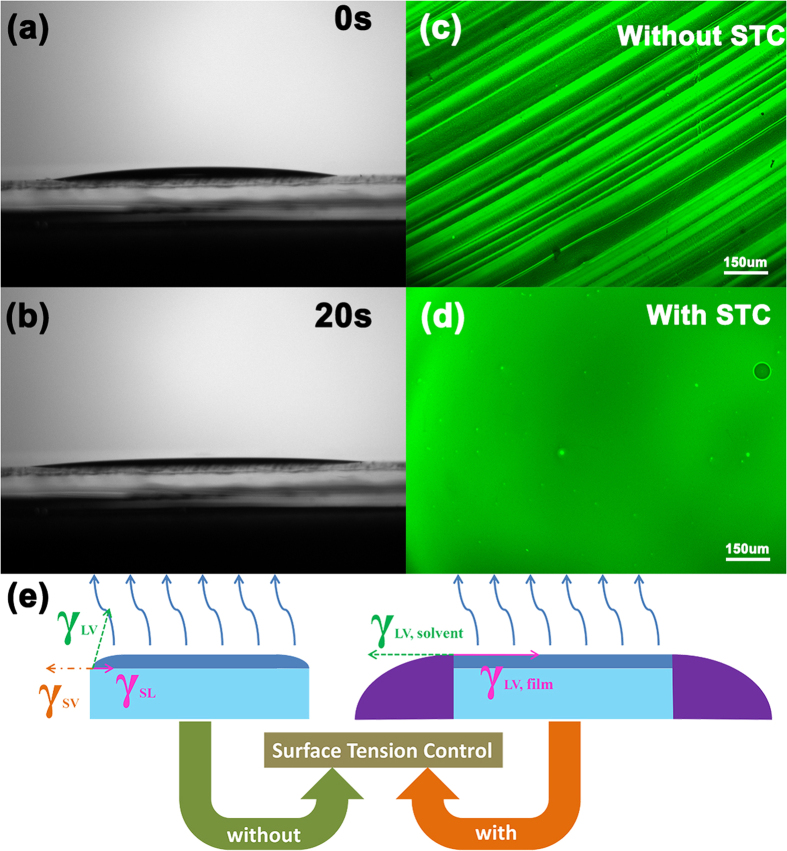
(**a**,**b**) The lateral static pictures of the toluene droplets after falling on the substrate and (**c**,**d**) the fluorescence microscopic image of the small molecular film fabricated without and with surface tension control process.

**Figure 6:**
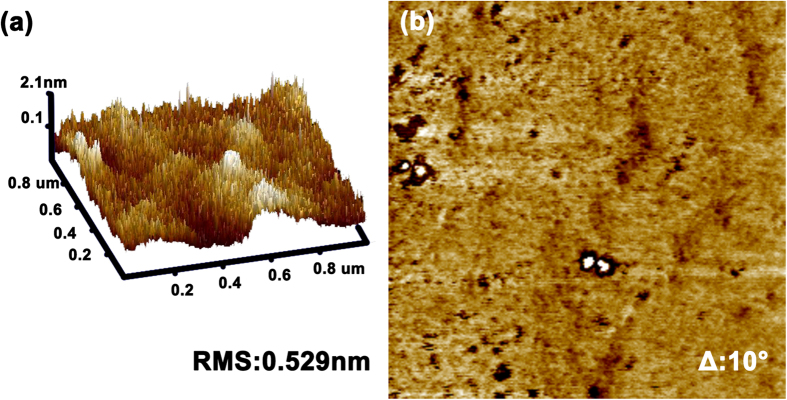


**Figure 7:**
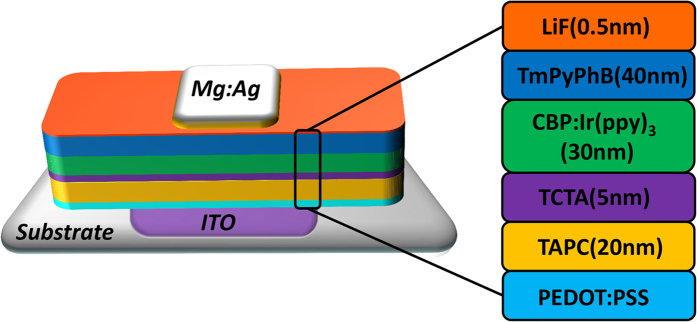


**Figure 8:**
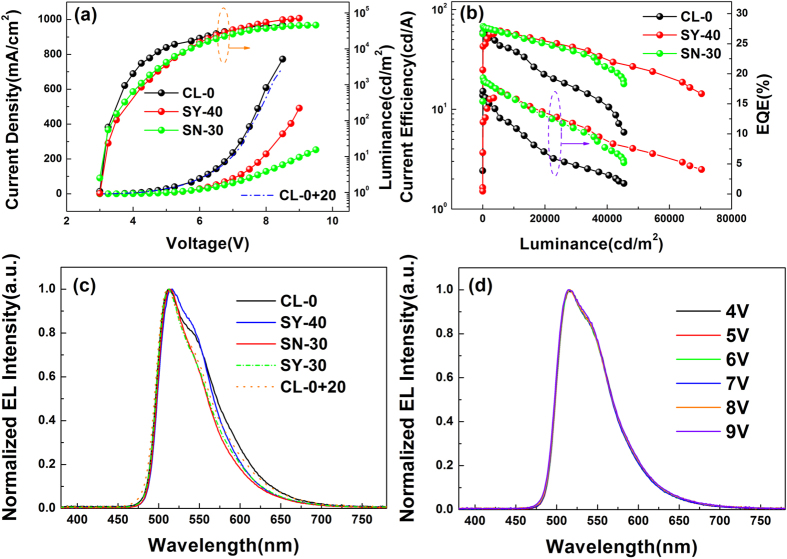
(**a**) The current density-voltage-luminance and (**b**) the current efficiency-luminance-external quantum efficiency characteristics of the CL-0, SY-40 and SN-30, (**c**) the EL spectra of CL-0, SY-40 and SN-30 at the luminance of 1000 cd/m^2^ and (**d**) the normalized EL spectra of SY-40 at different bias voltages.

**Figure 9:**
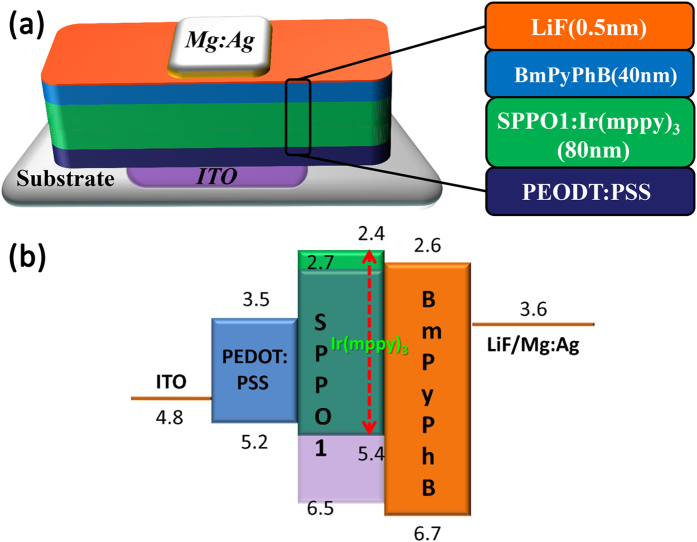
The (**a**) structure and (**b**) energy level schemes of the green OLEDs.

**Figure 10:**
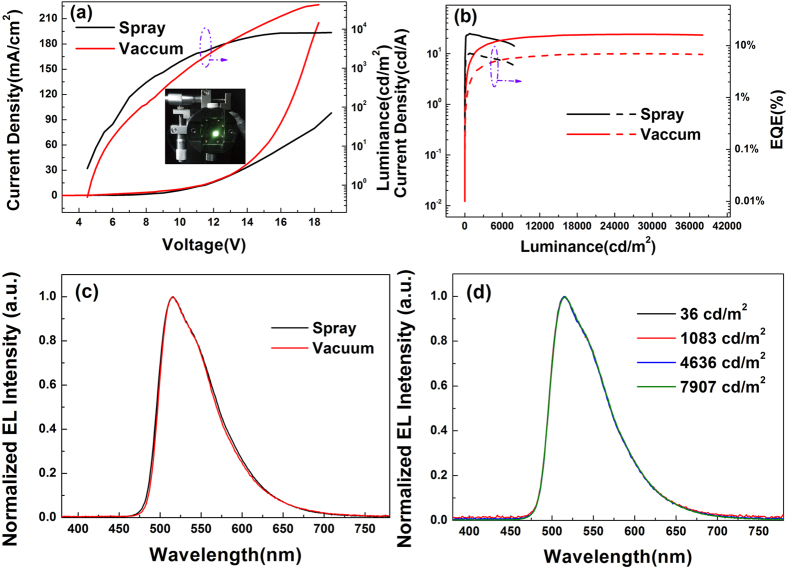
(**a**) The current density-voltage-luminance and (**b**) the current density-luminance-EQE characteristics of the green OLEDs, (**c**) the normalized EL spectra for green OLEDs at 1000 cd/m^2^ and (**d**) the normalized EL spectra for green OLEDs based on ultrasonic spray coating emitting layer, the insets is the picture of green OLEDs employing ultrasonic spray coating layer.
